# Cell cycle regulators control mesoderm specification in human pluripotent stem cells

**DOI:** 10.1074/jbc.RA119.008251

**Published:** 2019-09-12

**Authors:** Loukia Yiangou, Rodrigo A. Grandy, Anna Osnato, Daniel Ortmann, Sanjay Sinha, Ludovic Vallier

**Affiliations:** ‡ Wellcome–Medical Research Council Cambridge Stem Cell Institute, Anne McLaren Laboratory, University of Cambridge, Cambridge CB2 0SZ, United Kingdom; § Department of Surgery, University of Cambridge, Cambridge CB2 0QQ, United Kingdom; ¶ Department of Medicine, Division of Cardiovascular Medicine, University of Cambridge, Cambridge CB2 0QQ, United Kingdom; ‖ Wellcome Sanger Institute, Hinxton CB10 1SA, United Kingdom

**Keywords:** stem cells, differentiation, cell cycle, signaling, gene expression, cyclin-dependent kinase (CDK), pluripotency, embryo development, mesoderm, RB transcriptional corepressor 1 (RB1), tissue regeneration

## Abstract

The mesoderm is one of the three germ layers produced during gastrulation from which muscle, bones, kidneys, and the cardiovascular system originate. Understanding the mechanisms that control mesoderm specification could inform many applications, including the development of regenerative medicine therapies to manage diseases affecting these tissues. Here, we used human pluripotent stem cells to investigate the role of cell cycle in mesoderm formation. To this end, using small molecules or conditional gene knockdown, we inhibited proteins controlling G_1_ and G_2_/M cell cycle phases during the differentiation of human pluripotent stem cells into lateral plate, cardiac, and presomitic mesoderm. These loss-of-function experiments revealed that regulators of the G_1_ phase, such as cyclin-dependent kinases and pRb (retinoblastoma protein), are necessary for efficient mesoderm formation in a context-dependent manner. Further investigations disclosed that inhibition of the G_2_/M regulator cyclin-dependent kinase 1 decreases BMP (bone morphogenetic protein) signaling activity specifically during lateral plate mesoderm formation while reducing fibroblast growth factor/extracellular signaling-regulated kinase 1/2 activity in all mesoderm subtypes. Taken together, our findings reveal that cell cycle regulators direct mesoderm formation by controlling the activity of key developmental pathways.

## Introduction

Gastrulation represents an essential stage of early development when the three primary germ layers, endoderm, mesoderm, and ectoderm, are formed. Of particular interest for the current study, mesoderm specification occurs through a mesendoderm progenitor that is shared with endoderm. Mesoderm is then patterned in different subpopulations depending on their anteroposterior location in the primitive streak where a gradient of Nodal and BMP signaling and interplay with WNT and FGF^3^ leads to the formation of different cell types (1–3). At the very posterior end of the primitive streak, high BMP4 and low Nodal pattern extraembryonic mesoderm and the blood lineage, followed by anterior lateral plate mesoderm (1, 4, 5). The lateral plate mesoderm lineage gives rise to cardiovascular cell types such as smooth muscle cells and endothelial cells. At the more anterior part moderate levels of Nodal and BMP4 pattern cardiac mesoderm, which gives rise to cardiomyocytes, the main cell type constituting the heart (4, 6). In the middle anterior primitive streak, paraxial (presomitic) mesoderm is formed (7) that gives rise to bone, cartilage, and skeletal muscle and requires WNT signaling. Understanding the mechanisms directing the specification of these different types of mesoderm could have a broad implication for developmental biology but also in the context of diseases affecting stem cell differentiation. Nonetheless, studying these mechanisms at the molecular level remains challenging *in vivo* because of technical and ethical limitations in human.

Human pluripotent stem cells (hPSCs) provide a powerful alternative because they can proliferate almost indefinitely while maintaining the capacity to differentiate efficiently into the three germ layers (8). Thus, hPSCs have been used to uncover mechanisms directing germ layer specification (9–11). Of particular interest, studies have shown key functions for the cell cycle machinery in the specification of endoderm *versus* ectoderm and exit from the pluripotent state. Indeed, G_1_ and G_2_/M transition regulators have been shown to play a key role in pluripotency maintenance and cell fate decisions of hPSCs by controlling transcription factors, signaling pathways, and epigenetic regulators (12–16). More precisely, knockdown of CDK2 results in cell cycle arrest, decreased expression of pluripotency markers, and differentiation toward extraembryonic lineages (17). Similarly, abrogation of cyclin D1/2/3 results in loss of pluripotency and differentiation toward the mesendoderm lineage (13), indicating a direct role of cyclins and CDKs in the maintenance of pluripotency and cell identity. Furthermore, siRNA-mediated knockdown of CDK1 results in changes in cell morphology, decrease in pluripotency marker expression, accumulation of DNA damage, and mitotic deficiencies (18).

At the epigenetic level, histone modification H3K4me3 has been shown to be more abundant on developmental genes in the G_1_ phase of the cell cycle. Interestingly, the histone methyltransferase catalyzing this modification called MLL2 was also shown to be higher in the late G_1_ phase and enriched on promoters of the cell cycle regulated genes *SOX17* and *GATA6*. The recruitment of MLL2 on these developmental genes was shown to be mediated by phosphorylation of MLL2 by CDK2, establishing a role of cell cycle regulators in regulating epigenetic processes in hESCs (15).

Moreover, the cell cycle machinery and specifically the cyclin D–CDK4/6 complex has a vital role in guiding endoderm formation through regulation of the Nodal/Activin signaling pathway effector SMAD2/3. Cyclin D also acts as a transcriptional regulator independently of its role in the cell cycle and signaling regulation. ChIP-sequencing analyses have shown that cyclin D binds to and recruits transcriptional corepressor and coactivator complexes onto developmental gene loci, thus regulating their transcription and ultimately cell fate decisions (14).

Most of these studies, if not all, have been performed in the context of pluripotency, whereas the role of the cell cycle in guiding differentiation especially mesoderm specification is elusive. Here, we decided to address this question by taking advantage of protocols allowing differentiation of hPSCs into different mesoderm subtypes including lateral plate mesoderm (LPM), cardiac mesoderm (CM), and presomitic mesoderm (PSM) (19, 20). The corresponding culture system was used to investigate the role of G_1_ and G_2_/M cell cycle regulators in guiding mesoderm specification, especially the role of the cell cycle machinery in regulation of key developmental signaling pathways such as BMP, WNT, and FGF. Inhibition of both G_1_ and G_2_/M cell cycle regulators by small molecules blocked mesoderm subtype formation with different efficacy and in a context-dependent manner. Additional analyses into the molecular mechanisms responsible for this phenotype revealed that inhibition of the G_2_/M regulator CDK1 decreased BMP activity during LPM formation while reducing FGF/ERK activity in all mesoderm subtypes studied, thereby blocking differentiation. Our results demonstrate that cell cycle regulators are essential for the early stage of mesoderm formation and that this function is achieved through regulation of key developmental signaling pathways such as FGF/ERK. This knowledge will help to improve protocols for generating mesoderm cells *in vitro* and could also be relevant for the development of new therapies promoting tissue regeneration.

## Results

### Characterization of mesoderm subtypes generated from hPSCs

**Figure 1.**
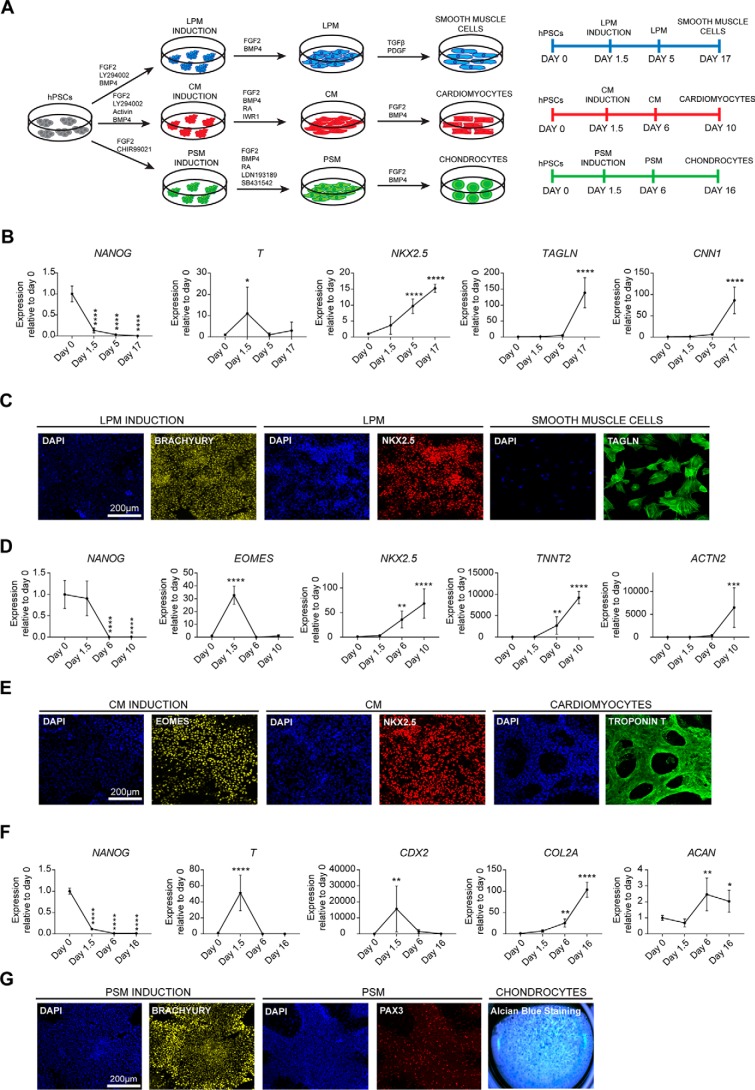
**Production of mesoderm subtypes from hPSCs.** *A*, schematic overview of the differentiation protocol. LPM was induced by FGF2, the PI3K inhibitor LY294002, and BMP4, and then SMCs were generated using TGFβ and PDGF-BB. CM was induced by Activin, FGF2, the PI3K inhibitor LY29400, and BMP4, and cardiac cells were obtained by inhibiting WNT signaling in the presence of BMP4, FGF2, and retinoic acid. PSM was induced by FGF2 and the WNT signaling agonist CHIR99021. PSM was then generated using FGF2, retinoic acid, and dual inhibition of TGFβ and BMP4 signaling using the small molecules SB431542 and LDN193189, respectively. Chondrocyte differentiation was induced by FGF2 and BMP4. *B*, RT-qPCR analysis for expression of pluripotency (*NANOG*) and mesoderm markers (*T*, *NKX2.5*, *TAGLN*, and *CNN1*) during SMC differentiation. *C*, immunostaining analysis for the expression of early mesoderm markers BRACHYURY, NKX2.5, and SMC marker TAGLN. *Scale bar*, 200 μm. *D*, RT-qPCR analysis for the expression of pluripotency (*NANOG*) and mesoderm markers (*EOMES*, *NKX2.5*, *TNNT2*, and *ACTN2*) during cardiomyocyte differentiation. *E*, immunostaining analysis for the anterior primitive streak marker EOMES, CM marker NKX2.5, and cardiomyocyte marker troponin T. *Scale bar*, 200 μm. *F*, RT-qPCR analysis for expression of pluripotency (*NANOG*) and mesoderm (*T*, *CDX2*, *COL2A1*, and *ACAN*) markers during chondrogenic differentiation. *G*, immunostaining analysis for the expression of early mesoderm marker BRACHYURY, PSM marker PAX3, and Alcian blue staining of chondrocytes differentiation. *Scale bar*, 200 μm. *Error bars* represent ± S.D. (*n* = 6). Ordinary one-way analysis of variance test followed by Dunnett's test for multiple comparisons was performed. *, *p* < 0.05; **, *p* < 0.01; ***, *p* < 0.001; ****, *p* < 0.0001.

In this study, we took advantage of established protocols for differentiating hPSCs into different mesoderm subtypes. Specifically, we took advantage of chemically defined culture conditions to drive differentiation of hPSCs into CM, LPM, and PM. These methods rely on growth factors known to direct mesoderm specification *in vivo* (20–22). As a result, hPSCs differentiation follows a natural path of development including the production of cells closely resembling cells arising along the anteroposterior axis of the primitive streak during development. In sum, hPSCs were induced to generate LPM, CM, and PSM mesoderm for 36 h followed by the addition of another mixture of growth factors and small molecules to generate functional cell types such as smooth muscle cells, cardiomyocytes, and chondrocytes (Fig. 1*A*) (20–22). During induction of all mesoderm subtypes, we observed a decrease in pluripotency marker expression such as *NANOG* and up-regulation of pan-mesoderm marker *BRACHYURY* (or *T*) (Fig. 1, *B–G*). LPM induction was confirmed by the increase in *NKX2.5* expression at day 5 (Fig. 1, *B* and *C*), and further differentiation toward smooth muscle cells was validated by the expression of calponin (*CNN1*) and transgelin (*TAGLN*) at day 17 (Fig. 1, *B* and *C*). During CM induction we observed expression of *EOMES* at day 1.5. CM identity was confirmed by the high expression of *NKX2.5* at day 6, whereas further differentiation resulting in beating cardiomyocytes expressed the genes *ACTN2* (coding for the microfilament protein α-Actinin) and *TNNT2* (coding for cardiac troponin T) (Fig. 1, *D* and *E*). Finally, PSM induction was associated with CDX2 expression at day 1.5 followed by chondrocyte differentiation as shown by the expression of the cartilage matrix proteins collagen 2a (*COL2A1*) and aggrecan (*ACAN*) (Fig. 1, *F* and *G*). Immunostaining analyses showed PAX3 expression in PSM, whereas Alcian blue staining confirmed production of proteoglycans such as aggrecan by terminally differentiated chondrocytes (Fig. 1*G*). Taken together, these results reinforce previous results by showing the robustness of our protocols to drive differentiation of hPSCs into different types of mesodermal progenitors.

### Inhibition of G_1_ and G_2_/M cell cycle regulators blocks induction of mesoderm subtypes in a context-dependent manner

**Figure 2.**
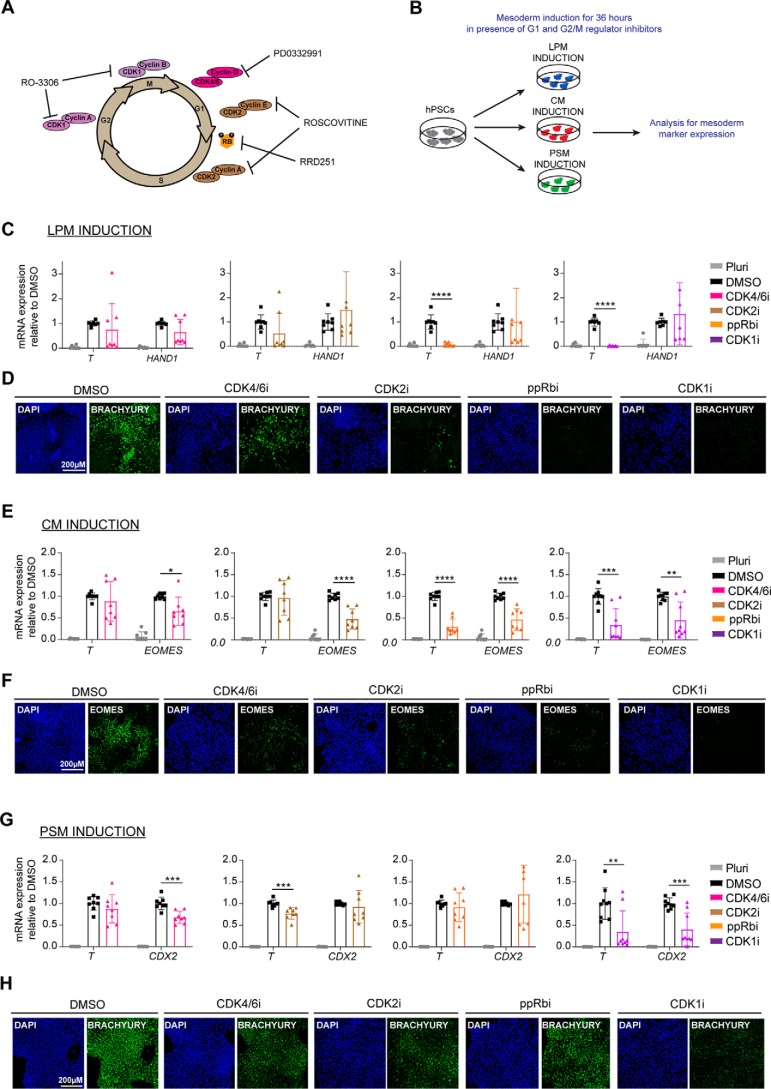
**Inhibition of cell cycle regulators blocks induction of mesoderm subtypes.** *A*, schematic showing the action of the different cell cycle regulator inhibitors used in the study. *B*, schematic overview of experimental setup to investigate the role of cell cycle regulators in mesoderm specification. For each experiment, hESCs were differentiated in the absence (DMSO) or presence of inhibitor. *C*, RT-qPCR analysis for the expression of mesoderm markers (*T* and *HAND1*) during LPM induction. *D*, immunostaining analysis for BRACHYURY expression during LPM induction. *Scale bar*, 200 μm. *E*, RT-qPCR analysis for the expression of mesoderm markers (*T* and *EOMES*) during CM induction. *F*, immunostaining analysis for EOMES expression during CM induction. *Scale bar*, 200 μm. *G*, RT-qPCR analysis for the expression of mesoderm markers (*T* and *CDX2*) during PSM induction. *H*, immunostaining analysis for the expression of BRACHYURY during PSM induction. *Scale bar*, 200 μm. *Error bars* represent ± S.D. of four independent experiments. Unpaired *t* test was performed. Differences between DMSO- and inhibitor-treated cells are shown. *, *p* < 0.05; **, *p* < 0.01; ***, *p* < 0.001; ****, *p* < 0.0001. *DAPI*, 4′,6′-diamino-2-phenylindole.

To explore the importance of cycle machinery in mesoderm specification, we next investigated the effect of the inhibition of G1 and G_2_/M regulators on differentiation. For that, we used small molecule inhibitors for CDK4/6 (PD-0332991), CDK2 (roscovitine), phosphorylation of retinoblastoma protein (RRD-251), and CDK1 (RO-3306; Fig. 2*A*). Of note, these small molecules are commonly used to study the function of cell cycle regulators in a diversity of systems. hESCs were induced to differentiate into the three mesoderm subtypes LPM, CM, and PSM in the presence of the cell cycle inhibitors for 36 h, and the resulting cells were harvested for gene expression and immunocytochemistry analyses (Fig. 2*B*). The cell cycle inhibitors did not cause cell death or cytotoxicity because morphology and cell density was not majorly affected (Fig. S1*A*). In the presence of the CDK1 inhibitor, slightly more cell death was observed as compared with the rest of the conditions tested (Fig. S1*A*). To exclude the possibility that any effects on differentiation are due to cytotoxicity of the small molecule cell cycle inhibitors, we assessed cell death after induction of the three mesoderm subtypes using annexin V and propidium iodide analysis. Overall, no big differences were observed in the percentage of dead cells after treatment with the small molecule cell cycle inhibitors as compared with DMSO-treated and pluripotent cells. The slightly higher percentage of dead cells observed upon CDK1 inhibitor treatment during CM and PSM induction is in agreement with the morphology and density observed in the culture (Fig. S1, *B–G*).

Concerning LPM differentiation, ppRb inhibition and CDK1 inhibition were associated with strong down-regulation of *T* expression (Fig. 2*C*). The inefficient expression of the master regulator *T* was also validated at the protein level where a reduction in expression of BRACHYURY was observed upon CDK4/6 and CDK2 inhibition, whereas inhibition of ppRb and CDK1 resulted to complete loss of BRACHYURY expression (Fig. 2*D*). Of note, down-regulation of the pluripotency markers *OCT4* and *NANOG* was not stopped, suggesting that inhibition of cell cycle regulators did not block differentiation of hPSCs (Fig. S2*A*). Interestingly, inhibition of CDK2 and ppRb caused more effective down-regulation of *OCT4* and *NANOG*, suggesting further regulation of not only differentiation marker but also pluripotency marker expression.

Similarly, CM specification was strongly altered upon inhibition of all cell cycle regulators, as shown by the reduction in *T* and *EOMES* expression (Fig. 2, *E* and *F*). Although no significant differences were observed in pluripotency markers, there was slightly higher expression of *OCT4* and *NANOG* during inhibition of CDK2, ppRb, and CDK1.

PSM induction was also inefficient upon inhibition of the cell cycle regulators. Expression of *T* was more significantly reduced upon inhibition of CDK2 and CDK1 and expression of *CDX2* upon inhibition of CDK4/6 and CDK1 (Fig. 2*G*). Immunostaining analysis for BRACHYURY expression confirmed RT-qPCR results (Fig. 2*H*). Expression of pluripotency genes did not significantly differ between DMSO and inhibitor-treated cells with the exception of CDK1 inhibition, which led to the maintenance of *OCT4* and *NANOG* expression during the differentiation process (Fig. S2*C*). This suggests that CDK1 could be necessary for the down-regulation of these genes and exit from pluripotency during PSM formation.

Taken together, these results show that cell cycle regulators are essential for mesoderm formation and subtype specification. Of note, the strong effect seen on *T* expression suggests that these regulators may be involved during the early stage of mesoderm specification corresponding to primitive streak formation. Furthermore, CDKs could also be necessary for the fine-tuning of pluripotency marker expression depending on the mesoderm subtype generated as suggested by the pattern of expression for *OCT4* and *NANOG*.

### Inhibition of G_1_ phase regulators does not significantly affect the activity of BMP, WNT, and FGF signaling pathways

**Figure 3.**
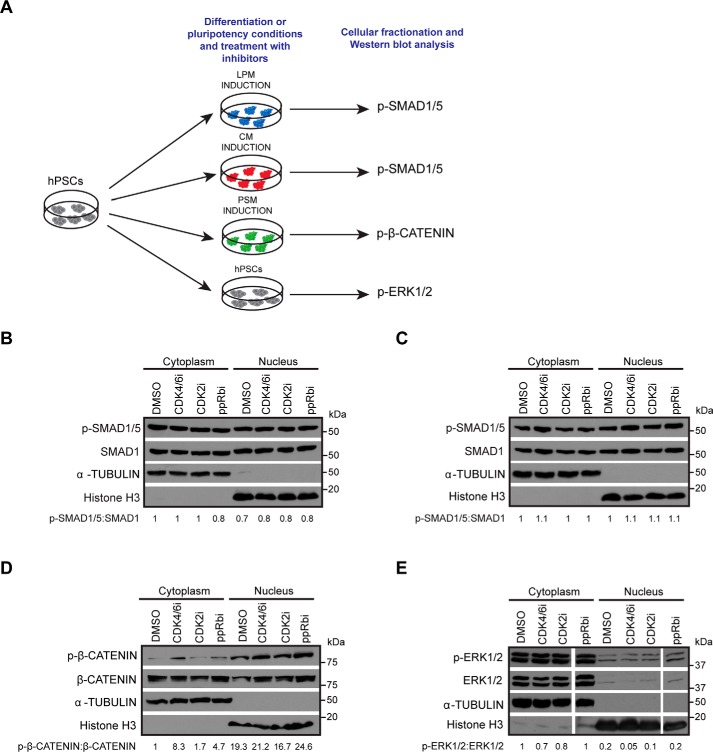
**BMP, WNT, and FGF signaling pathways are not affected by inhibition of G_1_ regulators.** *A*, schematic overview of experimental setup to determine activity of BMP, WNT, and FGF signaling upon treatment with G_1_ phase regulator inhibitors. *B* and *C*, Western blotting and densitometry analyses for phospho-SMAD1/5 and total SMAD1 to determine activity of BMP signaling in DMSO-treated *versus* cells treated with G_1_ regulator inhibitors during LPM (*B*) and CM induction (*C*). *D*, Western blotting and densitometry analyses for phospho–β-catenin and total β-catenin to determine activity of WNT signaling. *E*, Western blotting and densitometry analyses for phospho-ERK1/2 and total ERK1/2 to determine activity of FGF signaling. The blot represents samples run on the same gel. α-Tubulin and histone H3 were used as loading controls for the cytoplasm and the nuclear fractions, respectively.

Based on our previous results showing that CDK4/6 could control the activity of Activin/Nodal signaling (13), we hypothesized that regulators of the G_1_ phase, including CDK4/6, CDK2, and pRb, could control signaling pathways such as BMP, WNT, and FGF, which are known to direct mesoderm specification. To confirm this possibility, we studied the effect of G_1_ CDK inhibitors on the activity of these signaling pathways during the specification of different types of mesoderm. The effect on BMP signaling was analyzed in the context of LPM and CM formation because BMP4 is used to drive formation of these mesoderm subtypes, whereas the effect on WNT signaling was analyzed during PSM formation because CHIR99021, a WNT agonist, is driving this mesoderm formation. Finally, the effect on FGF signaling was primarily analyzed in pluripotent cells to avoid interference with other pathways. Indeed, FGF2 is important for all three mesoderm subtypes, LPM, CM, and PSM, and thus it was more informative to test the corresponding inhibitors in pluripotency conditions before further investigations during differentiation. Each mesoderm subtype was induced in the presence of the inhibitors for 36 h with the exception of FGF signaling, for which hESCs were grown for 12 h in the presence of inhibitors. Prior to harvesting for Western blotting analysis, the cells were freshly fed with media supplemented with the inhibitors and subsequently harvested for Western blotting analyses (Fig. 3*A*).

Despite several analyses and different conditions of treatment, inhibition of cell cycle regulators in these different culture conditions did not affect the levels of phospho-SMAD1/5 (Fig. 3, *B* and *C*), whereas minor differences were observed in the levels of phospho–β-catenin (Fig. 3*D*) among the different conditions tested. A slight increase in the levels of phospho–β-catenin was observed upon treatment with the CDK4/6 and ppRb inhibitors, suggesting that increased degradation of β-catenin can lead to lower WNT signaling, contributing to the down-regulation of *T* observed during PSM. Similarly to phospho-SMAD1/5, the level of phospho-ERK1/2 did not show major changes upon treatment with the small molecule inhibitors (Fig. 3*E*). Thus, G_1_ phase regulators appear to have only mild effects on BMP, WNT, and FGF signaling, and the pathways appear to be mostly active in the presence of the inhibitors, suggesting that the regulation of mesoderm formation by G_1_ regulators occurs through alternative mechanisms.

### Inhibition of CDK1 decreases the activity of BMP and FGF signaling pathways

**Figure 4.**
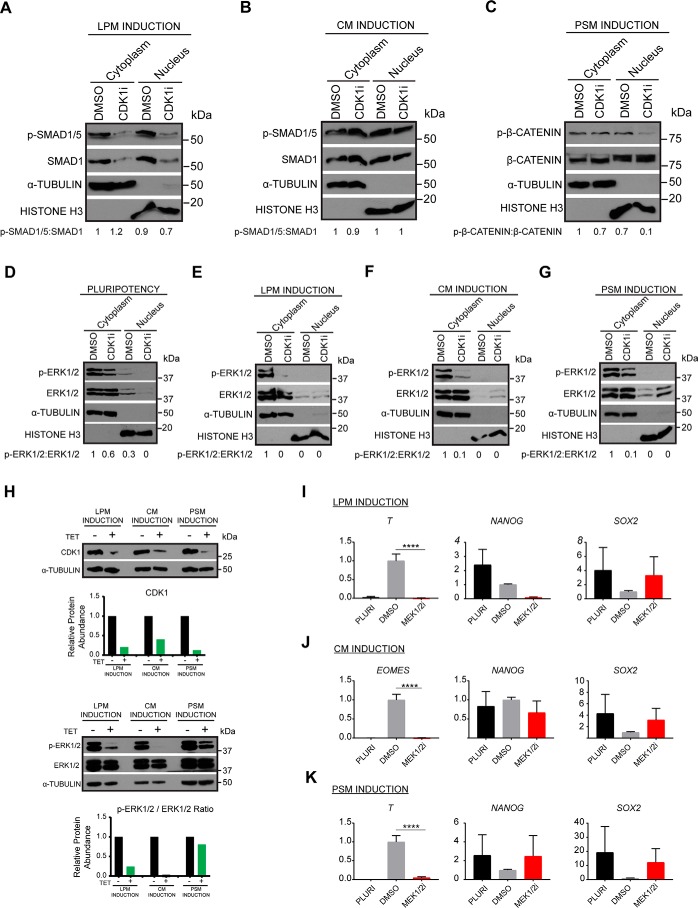
**Inhibition of CDK1 decreases BMP and FGF signaling pathway activity.** *A* and *B*, Western blotting and densitometry analyses for phospho-SMAD1/5 and total SMAD1 to determine activity of BMP signaling in DMSO-treated *versus* cells treated with the CDK1 inhibitor RO-3306 during LPM (*A*) and CM induction (*B*). *C*, Western blotting and densitometry analyses for phospho–β-catenin and total β-catenin to determine activity of WNT signaling in DMSO-treated *versus* cells treated with the CDK1 inhibitor RO-3306 during PSM induction. *D–G*, Western blotting and densitometry analyses for phospho-ERK1/2 and total ERK1/2 to determine activity of FGF signaling in pluripotency conditions (*D*) and during LPM (*E*), CM (*F*), and PSM induction (*G*). α-Tubulin and histone H3 were used as loading controls for the cytoplasm and the nuclear fractions, respectively. *H*, Western blotting analysis of CDK1 iKD cells. Pluripotent cells were treated with tetracycline for 4 days prior to induction of differentiation. Following 36 h of mesoderm subtype differentiation, the cells were harvested for analysis of phospho-ERK1/2 and CDK1 expression. Graphs show densitometric analysis of protein relative to loading control α-tubulin and normalized to treatments without tetracycline hydrochloride (*TET*). *I–K*, RT-qPCR analysis for expression of differentiation and pluripotency markers during induction of LPM (*I*), CM (*J*), and PSM (*K*). *Error bars* represent ± S.D. (*n* = 6). Ordinary one-way analysis of variance test followed by Tukey's test for multiple comparisons was performed. Differences between DMSO and inhibitor treated cells are shown. *, *p* < 0.05; **, *p* < 0.01; ***, *p* < 0.001; ****, *p* < 0.0001.

**Figure 5.**
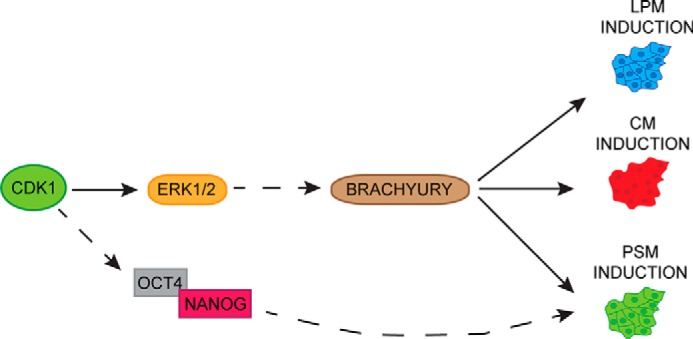
**Model depicting the role of CDK1 in mesoderm specification.** A schematic of main findings on role of CDK1 in mesoderm patterning is shown. CDK1 regulates FGF/ERK1/2 signaling to control *BRACHYURY* expression and subsequent mesoderm differentiation. Additionally, CDK1 could be directly or indirectly regulating expression of OCT4/NANOG to drive LPM formation.

We next investigated the effect of CDK1 inhibition on the same signaling pathways using similar conditions. Interestingly, CDK1 inhibitor strongly reduced the total level of SMAD1, resulting in a dramatic decrease in the levels of available phospho-SMAD1/5 (Fig. 4*A*) specifically during LPM differentiation but not induction of CM (Fig. 4*B*). Thus, CDK1 could control BMP signaling only in certain mesoderm subtypes. Further characterization showed that WNT signaling pathway was not affected by CDK1 (Fig. 4*C*), whereas FGF/ERK1/2 signaling pathway was severely down-regulated upon inhibition of CDK1 in pluripotency conditions (Fig. 4*D*) and even more strongly in all mesoderm subtypes. The most severe phenotype was observed in LPM and CM induction, in which the loss of phospho-ERK1/2 was almost complete (Fig. 4, *E* and *F*), whereas this reduction was less severe in PSM (Fig. 5*G*). To confirm these results, we decided to genetically validate this phenotype by knocking down CDK1 in hESCs. For that, we took advantage of the single-step optimized inducible knockdown (sOPTiKD) platform as previously described (24). The resulting CDK1 iKD-hESCs were treated with tetracycline for 5 days to induce the knockdown of CDK1 and subsequently differentiated into the three mesoderm subtypes in the presence of tetracycline. Western blotting analysis confirmed the efficient knockdown of CDK1 with 80, 60, and 90% decrease in expression in LPM, CM, and PSM, respectively (Fig. 4*H*, *first panel*). Western blotting analyses showed that decrease in CDK1 expression was associated with reduced ERK1/2 phosphorylation (Fig. 4*H*, *second panel*). The strongest effect was observed in LPM and CM induction, whereas reduction in PSM was less severe, recapitulating the phenotype obtained by inhibiting CDK1 with small molecule. Taken together, these results demonstrate that CDK1 is necessary for the activity of BMP and FGF signaling during mesoderm specification.

### Inhibition of FGF/ERK1/2 signaling blocks induction of all mesoderm subtypes

Our results also imply that ERK1/2 should be essential for mesoderm differentiation. To validate this hypothesis, we induced mesoderm subtype formation for 36 h in the presence of the ERK/MEK1/2 inhibitor PD0325901. Strikingly, presence of this small molecule resulted in the complete loss of expression of *T* during LPM (Fig. 4*I*) and PSM induction (Fig. 4*K*) and loss of *EOMES* during CM induction (Fig. 4*J*). Taken together, these results demonstrate that mesoderm subtype induction is controlled by the interplays between CDK1 and FGF/ERK1/2, which are necessary for the induction of key mesoderm markers but also the down-regulation of pluripotency factors.

## Discussion

In this study, we investigated the role of G_1_ and G_2_/M cell cycle regulators in the specification of mesoderm subtypes. We used pharmacological inhibition of CDK4/6, CDK2, and CDK1 using the small inhibitors PD-0332991, roscovitine, and RO-3306, respectively. Additionally, retinoblastoma protein (pRb) was maintained in its active state by inhibiting its phosphorylation with the small molecule RRD-251. Inhibition of each cell cycle regulator disrupted specific mesoderm differentiation. CDK4/6 appears to be necessary for expression of *T* in all the mesoderm subtypes, even though more significant changes were observed during ppRb and CDK1 inhibition. Interestingly, it was previously shown that CDK4/6 inhibits endoderm formation by blocking the nuclear import of SMAD2/3 (13). Thus, cell cycle regulators could be germ layer–specific and context-dependent. In our mesoderm differentiation model, only anterior primitive-like and subsequent cardiac mesoderm is induced in the presence of Activin, and high activity of SMAD2/3 is likely to perturb mesoderm induction. Thus, CDK4/6 could be necessary to safe guard mesoderm induction against ectopic Activin signaling activity.

Interestingly, CDK2 and phosphorylation of pRb also appear to be necessary for mesoderm specification. CDK2 is known to control phosphorylation of pRb during cell cycle progression, and thus both regulators could interact during differentiation (reviewed in Ref. 25). Accordingly, the most important effect of their inhibition is the loss of *T* expression specifically during LPM induction. These results also suggest that CDK2 and/or pRb could control the early stage of mesoderm induction corresponding to the primitive streak formation *in vivo*. Despite their functional importance, we were not able to establish a link between G_1_ phase regulators with signaling pathways controlling differentiation. Thus, CDK2/pRb could control alternative mechanisms necessary for mesoderm patterning. It has been previously proposed that cell cycle regulators and specifically CDK2 regulates epigenetic modifiers such as the MLL methyltransferase and guides it to developmental genes in the late G_1_ phase (15). This observation could also apply in our system and specifically it would be of interest to identify whether MLL2 is phosphorylated by CDK2 or CDK4/6 during mesoderm differentiation and whether there is recruitment of MLL2 to genes guiding mesoderm specification such as BRACHYURY, whose expression has been shown to rely on MLL2 (15).

In contrast to G_1_ cell cycle regulators, we observed a strong reduction in FGF/ERK1/2 signaling upon CDK1 inhibition in all mesoderm subtypes and a reduction in BMP4/SMAD1/5 signaling in during LPM induction. This observation is consistent with the interplay of FGF and BMP4 signaling in hESCs, which is known to be key for mesoderm differentiation (26, 27). Thus, CDK1 could be a link between the two signaling pathways, whose coordinated function is necessary for the expression of key mesoderm inducers such as *T*. FGF has been shown to regulate *T* expression in *Xenopus*, zebrafish, chick, and mouse embryos (28–33), and a correlation of FGF signaling and *T* expression was reported in human cancer cell lines (34). Thus, our results support *in vivo* and *in vitro* findings and suggest that a similar mechanism may be conserved in human stem cells.

Beyond differentiation markers, we also observed that inhibition of CDK2, ppRb, and CDK1 could affect pluripotency markers. The decrease in *OCT4* and *NANOG* expression was aggravated during LPM induction upon inhibition of CDK2 and ppRb and inhibited during PSM induction upon inhibition of CDK1. Thus, for some mesoderm subtypes, cell cycle regulators could fine-tune exit from pluripotency mechanisms in agreement with previous reports (12), whereas for other mesoderm subtypes, they could regulate the expression of pluripotency factors, guiding differentiation (20, 35–37).

Our results suggest an interesting link between pluripotency factor expression and up-regulation of differentiation genes. It has been shown that during BMP-induced differentiation, NANOG expression is prolonged (through ERK signaling), and NANOG knockdown causes loss of *T* expression in hESCs (26). Additionally, functional studies showed that loss of NANOG reduces expression of basal levels of primitive streak genes and loss of OCT4 results in *T* decrease in hESCs. Conversely, overexpression of NANOG was shown to increase levels of primitive streak genes (35). Moreover, a recent study has identified that ERK2 and CDK1 both phosphorylate NANOG (38). This is an intriguing correlation suggesting that the two kinases could regulate NANOG stability through phosphorylation in the G_2_/M phase in a cooperative manner to prevent overexpression or excessive decrease during the differentiation process. Considered together, these reports reinforce the data presented in the current study.

Importantly, validations of our finding *in vivo* remain challenging. Indeed, knockout of G_1_ cell cycle regulators results in viable embryos (39–43), whereas only the simultaneous knockout of CDK2 and CDK4 is embryonic lethal (44). These results suggest that G_1_ CDKs are not essential for embryo development at the early stages, possibly because of functional redundancy often witnessed among these regulators. On the other hand, knockout of CDK1 is essential for cell proliferation during early development, and its absence causes embryonic lethality at embryonic day 10.5 (45). CDK1 thus is considered a master regulator of cell cycle transition. Interestingly, the essential role of CDK1 has been further corroborated because it was shown to compensate for loss of CDK2, CDK4, and CDK6 in the cells by binding to cyclins E, A, B, and D (46, 47), highlighting its essential role throughout development.

Of note, inhibition of CDK1 in our system during the differentiation process could be blocking cell cycle progression, thus contributing to the inefficient differentiation of the cells. Although this is a possibility, studies from our lab have shown that blocking or slowing down cell cycle during differentiation using small molecules such as nocodazole does not prevent the induction of specific markers such as T.^4^

Importantly, we cannot exclude the possibility that CDK1 could control additional molecular mechanisms directing differentiation. Indeed, CDK1 is also known to control epigenetic modifiers such as the polycomb group protein EZH2 (enhancer of zeste homologue 2). This protein is a histone methyltransferase and catalyzes H3K27me3 leading to gene silencing and has been shown to have a role in maintenance of pluripotency in PSCs, among its multiple other roles (48–50). Intriguingly, CDK1 was shown to control EZH2 activity through phosphorylation (51), whereas EZH2 inhibition was shown to be important for mesoderm formation in hESCs (52). Considered together, these previous studies suggest that CDK1 could coordinate epigenetic modification with signaling pathway activity during progression of mesoderm differentiation.

Taken together, our results show that cell cycle regulators are essential for mesoderm differentiation and exit from pluripotency. The mode of action of these regulators appears to be specific for each mesoderm subtype depending on the regulation of T by pluripotency factors and FGF/ERK1/2 signaling (Fig. 5). These mechanisms represent an additional step to understand the precise interplays between cell cycle machinery and differentiation.

## Experimental procedures

### hESC culture and differentiation

H9 hESCs (WiCell, Madison, WI) were cultured on vitronectin-coated plates (10 μg/ml; Stem Cell Technologies) in E6 medium supplemented with 2 ng/ml TGF-β (R&D Systems) and 25 ng/ml FGF2 (Dr. Marko Hyvönen, Cambridge University). The cells were maintained by weekly passaging using 0.5 mm EDTA (Thermo Fisher Scientific). CDK1 iKD H9 hESCs cells were grown in the same conditions, supplemented with 1 μg/ml puromycin for selection of antibiotic resistant cells. CDK1 knockdown was induced by adding 2 μg/ml tetracycline hydrochloride (Sigma–Aldrich) to the culture medium 4 days prior to the start of differentiation.

The cells were differentiated into the three germ layers and functional cell types as previously described (20, 21). Mesoderm subtypes were generated in a two-step protocol. For LPM formation, the cells were cultured for 36 h in chemically defined media (CDM)-polyvinyl alcohol (PVA) supplemented with 20 ng/ml FGF2, 10 μm LY294002 (Promega), and 10 ng/ml BMP4 (R&D Systems). Subsequently the cells were cultured for 3 days in CDM-PVA supplemented with 20 ng/ml FGF2 and 50 ng/ml BMP4 changing medium every 2 days. For CM formation, the cells were cultured for 36 h in CDM-BSA (without insulin) supplemented with 20 ng/ml FGF2, 10 μm LY294002, 10 ng/ml BMP4, and 50 ng/ml Activin A (Dr. Marko Hyvönen, Cambridge University). Subsequently the cells were cultured for 4 days in CDM-BSA (without insulin) supplemented with 8 ng/ml FGF2, 10 ng/ml BMP4, 1 μm IWR1 (WNT signaling inhibitor; Tocris Bioscience), and 0.5 μm retinoic acid (Sigma–Aldrich), changing medium every 2 days. For PSM formation, the cells were cultured for 36 h in CDM-BSA (without insulin) supplemented with 20 ng/ml FGF2 and 8 μm CHIR99021 (WNT signaling activator; Tocris Bioscience). Subsequently cells were cultured for 4 days in CDM-BSA (with insulin) supplemented with 4 ng/ml FGF2, 1 μm retinoic acid, 0.1 μm LDN193189 (BMP signaling inhibitor; Sigma–Aldrich), and 10 μm SB431542 (TGF-β signaling inhibitor; Tocris Bioscience). For mesoderm differentiation cells were plated on gelatin and mouse embryonic fibroblast medium–coated plates. Functional differentiation of the mesoderm subtypes was performed as described in supporting text Small molecule cell cycle inhibitors used are listed in Table S1.

### RNA extraction, cDNA synthesis, and RT-qPCR

Total RNA was extracted using the GenElute^TM^ mammalian total RNA miniprep kit (Sigma–Aldrich) and the On-Column DNase I digestion set (Sigma–Aldrich) according to the manufacturer's instructions. RNA was reverse-transcribed using 250 ng of random primers (Promega), 0.5 mm dNTPs (Promega), 20 units of RNAseOUT, 0.01 m DTT, and 25 units of SuperScript II (all from Invitrogen). The resulting cDNA was diluted 30-fold for the qPCR. Quantitative PCR mixtures were prepared using the KAPA SYBR® FAST qPCR Master Mix (2×) kit (Kapa Biosystems), 4.2 μl of cDNA, and 200 nm of each of the forward and reverse primers. Samples were run on 384-well plates using the QuantStudio 12K Flex real-time PCR system machine and results analyzed using the delta-delta cycle threshold method (ΔΔCt). Expression values were normalized to the housekeeping gene *PBGD* (porphobilinogen deaminase). The primer sequences are listed in Table S2.

### Immunocytochemistry

The cells were fixed in 4% paraformaldehyde for 20 min at 4 °C followed by one wash with PBS. The cells were subsequently blocked and permeabilized at room temperature for 30 min in PBS with 4% donkey serum (Bio-Rad) and 0.1% Triton X-100 (Sigma–Aldrich). Primary antibodies were diluted in the same buffer and incubated with the cells overnight at 4 °C. After three washes with PBS, the cells were incubated with Alexa Fluor secondary antibodies for 1 h at room temperature protected from light. The cells were subsequently washed three times for 5 min with PBS, adding 5 μg/ml DAPI during the first wash to stain nuclei. The antibodies used are listed in Table S3.

### Alcian blue staining

Monolayer cultures of chondrocytes were fixed in 4% paraformaldehyde for 20 min at 4 °C. The cells were then washed with 0.5 n HCl and stained overnight with 0.25% (w/v) Alcian blue 8GX (Sigma–Aldrich) in 0.5 n HCl. Stained cells were visualized using a Leica dissecting microscope. Alcian blue dye was solubilized by overnight incubation with 8 m guanidine hydrochloride (Sigma–Aldrich) and quantified by absorbance at 595 nm using a spectrophotometer.

### Generation of CDK1 inducible knockdown line

Knockdown for CDK1 was performed using the single optimized inducible knockdown method as previously described (23, 24). Multiple shRNAs for the CDK1 gene were obtained from the validated shRNA database at Sigma–Aldrich. Briefly, shRNAs were introduced in the psOPTiKD plasmid between the BglII and SalI-HF sites. The psOPTiKD-shCDK1 vector was targeted to the AAVS1 locus by using 6 μg of each of the following vectors: psOPTiKD-shCDK1, pZFN.AAVS1-KKR, and pZFN.AAVS1-ELD. H9 hESCs were nucleofected using the Lonza P3 primary Cell 4D nucleofector X kit, and monoclonal colonies were selected for 7–10 days with 1 μg/ml of puromycin (Sigma–Aldrich). Tetracycline hydrochloride (Sigma–Aldrich) was used at 1 μg/ml to induce the expression of the shRNA. Knockdown of CDK1 was confirmed by Western blotting using anti-CDK1 antibody (Abcam, Ab133327). The shRNA sequences are listed in Table S4.

### Cellular fractionation and Western blotting

The cells were washed once with PBS and harvested with cell dissociation buffer (Gibco) for 10 min at 37^°^C. After one wash with cold 1% BSA-PBS, the pellets were collected by centrifugation at 4 °C and 300 × *g* for 3 min. For isolation of the cytoplasmic fraction, the pellets were resuspended in six times packed cell volume equivalent of isotonic lysis buffer (10 mm Tris-HCl, 3 mm CaCl, 2 mm MgCl_2_, 0.32 m sucrose, pH 7.5) supplement with protease and phosphatase inhibitors (Roche) and incubated for 12 min on ice. 0.3% Triton X-100 (Sigma–Aldrich) was added for a further 3 min, and samples were centrifuged at 1,800 rpm for 5 min at 4 °C. The supernatant (cytoplasmic fraction) was collected in a fresh chilled tube. The nuclear pellet was washed once with isotonic lysis buffer and centrifuged at 4,000 rpm for 3 min at 4 °C. The nuclear pellet was resuspended in two times the original packed cell volume equivalent of nuclear lysis buffer (50 mm Tris-HCl, pH 7.5, 100 mm NaCl, 50 mm KCl_2_, 1 mm EDTA, 10% glycerol, 0.3% Triton X-100) supplemented with protease and phosphatase inhibitors (Roche), homogenized with a pellet pestle (Kimble Chase), and incubated for 30 min on ice. Subsequently, 125 units of benzonase nuclease (Sigma–Aldrich) was added to the lysates and incubated at room temperature for 45 min to remove nucleic acids. For extraction of whole cell lysate, the cells were lysed with radioimmune precipitation assay lysis buffer (50 mm Tris-HCl, 150 mm NaCl, 1% Triton X-100, 0.5% sodium deoxycholate, 0.1% SDS, pH 8.0). Protein was quantified using the Pierce BCA protein assay kit (Thermo Fisher Scientific) according to the manufacturer's instructions. Lysates were prepared for Western blotting by adding 1× NuPAGE LDS sample buffer (Thermo Fisher Scientific) with 1% β-mercaptoethanol and incubated at 95 °C for 5 min. Lysates (10–35 μg of protein) were electrophoresed on 12% NuPAGE Bis-Tris precast gels (Thermo Fisher Scientific) with NuPAGE MOPS SDS running buffer (Thermo Fisher Scientific). For identification of the size of the target protein, Precision Plus Protein Ladder was used (Bio-Rad). Protein was transferred on PVDF membranes (Bio-Rad) by liquid transfer using NuPAGE transfer buffer (Thermo Fisher Scientific). Membranes were blocked using 4% nonfat dried milk in TBS and 0.1% Tween (TBST buffer) for 30 min and probed with primary antibody overnight at 4 °C in TBST. Following three washes of 5 min each with TBST, membranes were incubated with horseradish peroxidase–conjugated secondary antibody for 1 h at room temperature in TBST. Membranes were then washed three times for 5 min with TBST and incubated with Pierce ECL Western blotting substrate and exposed to X-ray Super RX films (Fujifilm). In cases where Western blotting membranes were incubated with more than one antibodies, the membranes were stripped using mild stripping buffer (1.5% glycine, 0.1% SDS, 1% Tween 20, pH 2.2). The membranes were incubated twice for 10 min with the stripping buffer, twice for 10 min with PBS, and twice for 5 min with TBST prior to blocking and incubation with primary antibody. The antibodies used are listed in Table S5.

## Author contributions

L. Y. and L. V. conceptualization; L. Y. data curation; L. Y. formal analysis; L. Y. validation; L. Y., R. A. G., A. O., and D. O. investigation; L. Y. visualization; L. Y. and L. V. methodology; L. Y. writing-original draft; L. Y., R. A. G., S. S., and L. V. writing-review and editing; R. A. G. and L. V. resources; S. S. and L. V. supervision; S. S. and L. V. project administration; L. V. funding acquisition; R. A. G. generation and provision of CDK1 inducible knockdown line.

## Supplementary Material

Supporting Information

## Footnotes

4 R. A. Grandy *et al.*, unpublished observations.

## Footnotes

3 The abbreviations used are:

FGF: fibroblast growth factor
hPSC: human pluripotent stem cell
CDK: cyclin-dependent kinase
ERK: extracellular signaling-regulated kinase
LPM: lateral plate mesoderm
CM: cardiac mesoderm
PSM: presomitic mesoderm
qPCR: quantitative PCR
sOPTiKD: single-step optimized inducible knockdown
pRb: retinoblastoma protein
ppRb: phosphorylated retinoblastoma protein.
