# Supplementary material for: Cell cycle regulators control mesoderm specification in human pluripotent stem cells
Source: J Biol Chem. 2019 Sep 12;294(47):17903–14. doi: 10.1074/jbc.RA119.008251 (PMC6879335; doi:10.1074/jbc.RA119.008251)
Supplement: Supporting Information [file supp_294_47_17903__index.html]

Cell cycle regulators control mesoderm specification in human pluripotent stem cells — Cell cycle regulators control mesoderm specification — Supporting Information 

# Cell cycle regulators control mesoderm specification in human pluripotent stem cells

## Supporting Information

- Cell cycle regulators control mesoderm specification in human pluripotent stem cells - Supporting Information
