# Supplementary material for: Cell cycle regulators control mesoderm specification in human pluripotent stem cells
Source: J Biol Chem. 2019 Sep 12;294(47):17903–14. doi: 10.1074/jbc.RA119.008251 (PMC6879335; doi:10.1074/jbc.RA119.008251)
Supplement: Supporting Information [file supp_RA119.008251_144064_2_supp_391989_pxkdl2.pdf]

Cell cycle regulators control mesoderm specification in human pluripotent stem cells

Loukia Yiangou, Rodrigo A. Grandy, Anna Osnato, Daniel Ortmann, Sanjay Sinha, and Ludovic Vallier

Material Included

- Results: Figure S1-S2
- Experimental procedures

Results

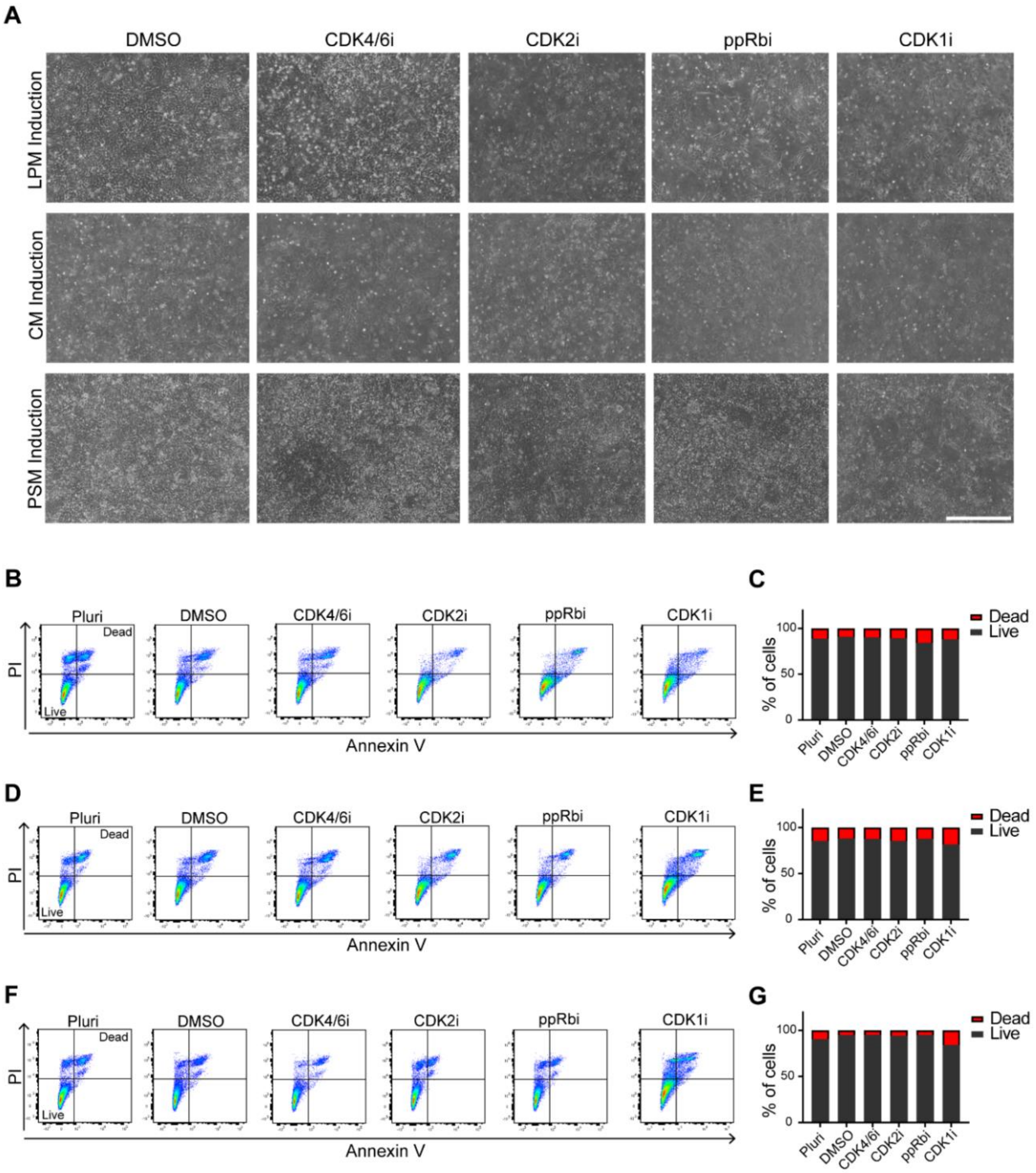

FIGURE S1. **Treatment with small molecule cell cycle inhibitors does not cause apoptosis and cell death.** *A*, Brightfield images of cells following mesoderm subtype induction in the presence of the small molecule cell cycle inhibitors. Scale bar: 1000µm. *B*, Flow cytometry analysis for Annexin V and Propidium iodide (PI) positive cells and *C*, Bar graph summarising flow cytometry results during LPM induction. *D*, Flow cytometry analysis for Annexin V and Propidium iodide (PI) positive cells and *E*, Bar graph summarising flow cytometry results during CM induction. *F*, Flow cytometry analysis for Annexin V and Propidium iodide (PI) positive cells and *G*, Bar graph summarising flow cytometry results during PSM induction.

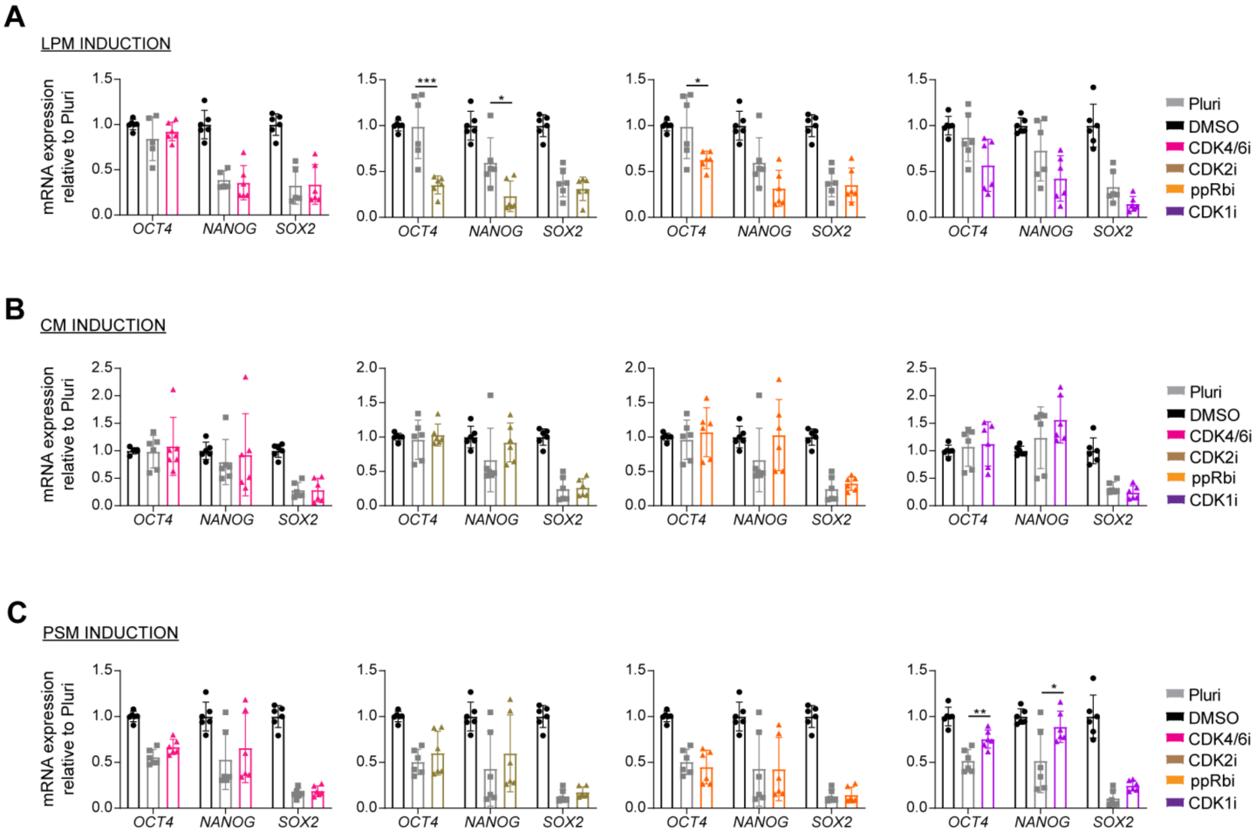

FIGURE S2. **Inhibition of G1 and G2/M cell cycle regulators affects pluripotency gene expression.** *A*-*C*, RT-qPCR analysis for expression of the pluripotency markers *OCT4*, *NANOG* and *SOX2* and *HAND1* during LPM induction (*A*), CM induction (*B*) and PSM induction (*C*) upon treatment with inhibitors of G1 and G2/M cell cycle regulators. Error bars represent ±SEM (n=3). Ordinary one-way ANOVA test followed by Tukey's test for multiple comparisons was performed. Differences between DMSO and inhibitor treated cells are shown *p<0.05, **p< 0.01, ***p<0.001, ****p<0.0001.

Experimental procedures

Smooth Muscle Cell Differentiation

For smooth muscle cell formation, LPM cells were dissociated with TrypLE Express (Life Technologies) for 5 minutes at 37ºC, washed once with CDM-PVA and centrifuged at 200g for 3 minutes. Cells were seeded on gelatin and MEF medium coated plates at a density of $2.6x10^4$ cells/cm^2 in CDM-PVA supplemented with 10ng/ml PDGF-BB (Peprotech) and 2ng/ml TGF-β (Peprotech) for 12 days. Media was changed every two days and cells split when confluent at a 1:2 ratio usually on day 3 or day 6.

Cardiomyocyte Differentiation

Following cardiac mesoderm formation, cells were cultured for two days in CDM-BSA (with insulin) supplemented with 8ng/ml FGF2 and 10ng/ml BMP4 (R&D) and subsequently fed every two days with CDM-BSA (with insulin). Onset of beating was observed on day 7-9 of differentiation.

Chondrocyte Differentiation

Following presomitic mesoderm formation, cells were cultured in CDM-BSA (with insulin) supplemented with 8ng/ml FGF2 and 10ng/ml BMP4 (R&D) for 10 days, changing media every two days.

Cells were treated with the small molecule inhibitors presented in table S1 and a number of different doses were tested. The inhibitors used are as follows:

Table S1. Small molecule cell cycle inhibitors used

**Inhibitor**	**Concentrations used**	**Catalogue Number**	**Supplier**
PD-0332991	5µM	S1116	Selleckchem
RO-3306	10µM	S7747	Selleckchem
Roscovitine	4µM	R7772	Sigma-Aldrich
RRD-251	10µM	R7532	Sigma-Aldrich

Table S2. qPCR primers used

**Gene**	**Forward Primer (5'-3')**	**Reverse Primer (5'-3')**
ACAN	CCCCTGCTATTTCATCGACCC	GACACACGGCTCCACTTGAT
ACTN2	CAAACCTGACCGGGGAAAAAT	CTGAATAGCAAAGCGAAGGATGA
CDX2	GGCAGCCAAGTGAAAACCAG	TTCCTCTCCTTTGCTCTGCG
CNN1	GTCCACCCTCCTGGCTTT	AAACTTGTTGGTGCCCATCT
COL2A1	TGGACGCCATGAAGGTTTTCT	TGGGAGCCAGATTGTCATCTC
EOMES	ATCATTACGAAACAGGGCAGGC	CGGGGTTGGTATTTGTGTAAGG
HAND1	GTGCGTCCTTTAATCCTCTTC	GTGAGAGCAAGCGGAAAAG
MIXL1	GGTACCCCGACATCCACTTG	TAATCTCCGGCCTAGCCAAA
NANOG	CATGAGTGTGGATCCAGCTTG	CCTGAATAAGCAGATCCATGG

NKX2.5	GAGCCGAAAAGAAAGCCTGAA	CACCGACACGTCTCACTCAG
PBGD	GGAGCCATGTCTGGTAACGG	CCACGCGAATCACTCTCATCT
T (BRACHYURY)	TGCTTCCCTGAGACCCAGTT	GATCACTTCTTTCCTTTGCATCAAG
TAGLN	TCTTTGAAGGCAAAGACATGG	TTATGCTCCTGCGCTTTCTT
TNNT2	ACAGAGCGGAAAAGTGGGAAG	TCGTTGATCCTGTTTCGGAGA

Table S3. Antibodies used for immunocytochemistry analysis

**Antibody**	**Species**	**Dilution**	**Catalogue Number**	**Manufacturer**
BRACHYURY	Goat	1:200	AF2085	R&D Systems
CARDIAC TROPONIN T	Rabbit	1:500	Ab45932	Abcam
EOMES	Mouse	1:200	MAB6166	R&D Systems
NKX2.5	Goat	1:500	sc-14033	Santa Cruz Biotechnology
PAX3	Mouse	1:100	-	Dev. Hybridoma Bank
SM22a/TAGLN	Rabbit	1:1,000	ab-14106	Abcam
Alexa Fluor 488 donkey anti-goat	-	1:1,000	A11055	Invitrogen
Alexa Fluor 488 donkey anti-mouse	-	1:1,000	A21202	Invitrogen
Alexa Fluor 488 donkey anti-rabbit	-	1:1,000	A21206	Invitrogen
Alexa Fluor 647 donkey anti-goat	-	1:1,000	A21447	Invitrogen
Alexa Fluor 647 donkey anti-mouse	-	1:1,000	A31571	Invitrogen
Alexa Fluor 647 donkey anti-rabbit	-	1:1,000	A31573	Invitrogen

Table S4. CDK1 shRNA sequences

**Top Oligo**	**Bottom Oligo**
GATCCCGCTGTACTTCGTCTTCTAATTCTCGAGAATTAGAAGACGAAGTACAGCTTTTTTG	TCGACAAAAAAGCTGTACTTCGTCTTCTAATTCTCGAGAATTAGAAGACGAAGTACAGCGG

Table S5. Primary antibodies used for Western blot analysis

Antibody	Species	Dilution	Catalogue Number	Manufacturer
Phospho-Smad1/5 (Ser463/465) (41D10)	Rabbit (Biotinylated)	1:500	9516S	Cell Signalling Technology
Smad1	Rabbit	1:500	9743S	Cell Signalling Technology
Phospho-β-catenin (Ser33/37/Thr41)	Rabbit	1:1,000	9561S	Cell Signalling Technology
β-catenin	Goat	1:1,000	AF1329	R&D Systems
Phospho-p44/p42 MAPK (Erk1/2) (Thr202/Tyr204)	Rabbit	1:1,000	9101S	Cell Signalling Technology
p44/42 MAPK (Erk1/2)	Rabbit (Biotinylated)	1:1,000	9102S	Cell Signalling Technology
A-tubulin	Mouse	1:40,000	T9026	Sigma-Aldrich
B-actin	Mouse	1:40,000	A2228	Sigma-Aldrich
Anti-CDK1	Rabbit	1:2,000	Ab133327	Abcam
Histone H3	Rabbit	1:40,000	H0164	Sigma-Aldrich
Anti-goat HRP	-	1:10,000	A5420	Sigma-Aldrich
Anti-rabbit HRP	-	1:10,000	A0545	Sigma-Aldrich
Anti-mouse HRP	-	1:10,000	A2554	Sigma-Aldrich
Streptavidin-HRP	-	1:2,000	3999S	Cell Signalling Technology
